# Dissociated Response in Metastatic Cancer: An Atypical Pattern Brought Into the Spotlight With Immunotherapy

**DOI:** 10.3389/fonc.2020.566297

**Published:** 2020-09-18

**Authors:** Olivier Humbert, David Chardin

**Affiliations:** ^1^Department of Nuclear Medicine, Centre Antoine-Lacassagne, Université Côte d'Azur, Nice, France; ^2^TIRO-UMR E 4320, Université Côte d'Azur, Nice, France

**Keywords:** dissociated, response, immunotherapy, metastatic cancer, heterogeneous response, imaging

## Abstract

When evaluating metastatic tumor response to systemic therapies, dissociated response is defined as the coexistence of responding and non-responding lesions within the same patient. Although commonly observed on interim whole-body imaging, the current response criteria in solid cancer do not consider this evolutive pattern, which is, by default, assimilated to progression. With targeted therapies and chemotherapies, dissociated response is observed with different frequencies, depending on the primary cancer type, treatment, and imaging modality. Because FDG PET/CT can easily assess response on a lesion-by-lesion basis, thus quickly revealing response heterogeneity, a PET/CT dissociated response has been described in up to 48% of women treated for a metastatic breast cancer. Although some studies have underlined a specific prognostic of dissociated response, it has always ended up being described as an unfavorable prognostic pattern and therefore assimilated to the “Progressive Disease” category of RECIST/PERCIST. This dichotomous imaging report (response vs. progression) provides a simple information for clinical decision-support, which probably explains the relatively low consideration for the dissociated response pattern to chemotherapies and targeted therapies until now. With immune checkpoint inhibitors, this paradigm is quickly changing. Dissociated response is observed in around 10% of advanced lung cancer patients and appears to be associated to treatment efficiency. Indeed, for this subset of patients, a clinical benefit of immunotherapy and favorable prognosis are usually observed. This specific pattern should therefore be considered in the future immunotherapy-adapted criteria for response evaluation using CT and PET/CT, and specific clinical managements should be evaluated for this response pattern.

## Introduction

**Figure 1:**
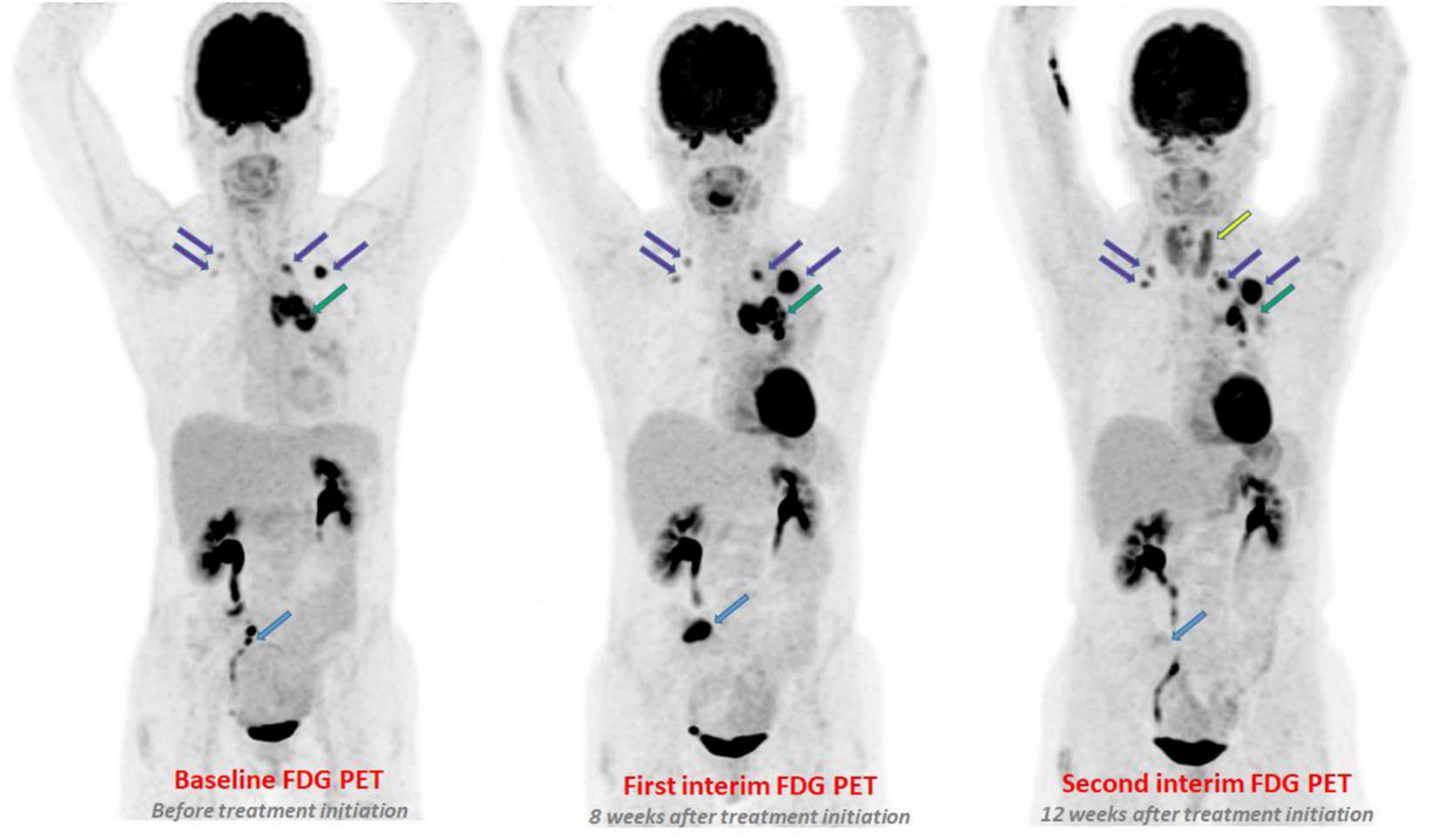
A 60-year-old women with metastatic adenocarcinoma treated with pembrolizumab. On first PETinterim at 8 weeks of treatment (three cycles), the maximum intensity projection image (MIP) showed a metabolic progression of a bone lesion of the sacrum (light blue arrow), of a left mediastino-hilar mass (green arrow) and of lung nodules (purple arrows). The subsequent PETinterim, performed at 12 weeks of treatment (5 cycles), showed a dissociated response with disappearance of the bone lesion of the sacrum (light blue arrow), clear metabolic regression of the left mediastino-hilar mass (green arrow) and metabolic progression of the lung nodules (purple arrows). A thyroiditis was also observed (yellow arrow). Because, the clinical status of the patient remained stable, the treatment was continued and the patient finally obtained a prolonged durable clinical benefit of immunotherapy (16 months of treatment).

When evaluating tumor response to systemic therapies in the metastatic setting, dissociated response (also termed mixed response, or heterogeneous response) is usually defined as the coexistence of responding and non-responding lesions within the same patient (Figure 1). Although dissociated response is a commonly observed evolutive pattern to systemic therapies, little is known about the biological specifications or the prognostic significance of this atypical pattern. This review aims to report what we already know about dissociated response in the setting of targeted therapies and chemotherapies, and highlight the new knowledge gained with the appearance of immunotherapy.

## Physio-Pathological Hypotheses

A combination of factors may explain the underlying biological mechanisms of a dissociated tumor response.

Firstly, because genomic instability occurs during the clonal evolution of solid cancers, multiple coexisting metastases can arise from genetically different tumor clones (1, 2). Indeed, cancerous cells not only undergo clonal evolution from a single progenitor cell into more aggressive and therapy resistant cells, but also exhibit branched evolution, whereby each tumor develops and preserves multiple distinct sub-clonal populations (2). This genotypic and phenotypic heterogeneity is an unfavorable prognostic factor for survival and can explain a dissociated response, particularly when using targeted therapies due to their selective pressure on tumor evolution (1–3).

Secondly, micro-environmental differences among metastatic sites can also induce heterogeneous responses. For instance, systemic therapies have a lower diffusion in bone tissue which is due to highly complex and variable interactions between tumor cells, bone cells and the bone matrix and can lead to a lower efficiency. Concerning brain metastasis, the blood-brain barrier can be a critical obstacle to the diffusion of certain drugs even though they are effective in other organs. Moreover, the heterogeneity of the immune environment of the lesions can actively influence therapeutic response and therefore explain different responses across lesions (4).

Some authors have suggested exercising caution when observing a dissociated response (5). Indeed, some unrelated processes may be pitfalls and erroneously mimic a mixed response. These pitfalls include: Synchronous neoplasms, inflammatory processes observed on FDG PET/CT (fat necrosis, diverticulitis…) or treatment-related effects (radiation-induced inflammation…). Thus, Clark et al. have defined an apparent dissociated response on FDG PET/CT to be a red flag that should lead to reconsider whether all findings are metastases of the same cancer. But we believe that radiologists and nuclear physicians are aware of this risk and that this pitfall rarely explain the heterogeneous response pattern assessed on PET/CT.

## Impact of the Imaging Modality on Response Assessment

What is striking concerning the few papers that have studied dissociated response is that most were performed using FDG PET/CT. At baseline, PET/CT is a highly sensitive imaging technique that can quickly depict the whole metastatic pool of lesions since hypermetabolic lesions appear with a high contrast. After initiating systemic treatment, FDG-PET/CT provides a whole-body quantitative assessment of treatment-induced changes in tumoral glycolysis and can be used to assess response on a lesion-by-lesion basis early on. Thus, a unique lesion with discordant evolution within the whole tumor load can easily be detected, revealing response heterogeneity (6–8). This explains why, even though the dissociated response pattern can also be assessed with CT, it is mostly described in PET/CT studies.

It's worth noting that dissociated response can be observed at any time during the imaging follow-up of a patients treated with systemic therapies. Despite the fact that most studies have evaluated dissociated response occurrence during the first 2 or 3 months of treatment (Table 1), it is currently unknown if it is more likely to be observed at early or late evaluation time points.

**Table 1:** Summary of publications concerning dissociated response in solid cancers.

**References**	**First author**	**No. of patients**	**Primary cancer**	**Study design**	**Drug class**	**Imaging modality**	**% of DR**	**Prognostic significance of DR**	**Timing after treatment initiation**
**CHEMOTHERAPIES AND TARGETED THERAPIES IN SOLID CANCERS**									
(9)	M. S. Carlino	23	Melanoma (≥2 lesions)	Monocentric Prospective	BRAF inhibitor	PET/CT	26%	DR = Shorter TTP than homogeneous responder	Day 15
(7)	A. Hendlisz	92	Metastatic colorectal cancer	Multicentric Prospective	Sorafenib + Capecitabine.	PET/CT	32%	The presence of at least one non-responding lesion is associated with a poorer out-come	Day 21
(10)	E. J. van Helden	9	Advanced KRAS wild-type colorectal adenocarcinoma	Monocentric Prospective	EGFR inhibitor	PET/CT	43%	-	Week 4
(11)	Y. Lee	68	Advanced NSCLC (IIIB-IV)	Monocentric Retrospective	EGFR-TKIs	CT and others	32%	-	Mostly at Week 8
(3)	Z. Y. Dong	246	Advanced or metastatic NSCLC	Monocentric Retrospective	Chemotherapy or EGFR-TKIs	PET/CT	21%	DR = an independent unfavorable prognostic factor for PFS and OS	NK
(12)	V. Huyge	25	Bone-dominant metastatic breast cancer	Monocentric Retrospective	Different systemic therapies	PET/CT	48%	TTP tends to be higher in patients with DR compared to those with a homogeneous non-response.	≤12 months
**IMMUNOTHERAPIES IN SOLID CANCER**									
(13)	M. Tazdait	160	Advanced NSCLC	Monocentric Retrospective	PD-1/L1 inhibitors	CT	7.5%	DR = better overall survival than true progression	Mostly at Week 6
(14)	T. Tozuka	62	Advanced NSCLC	Monocentric Retrospective	PD-1/L1 inhibitors	CT	9%	DR = favorable prognosis compared to homogeneous progression	≤2 months
(15)	O. Humbert	50	Advanced NSCLC (III-IV)	Monocentric Prospective	PD-1 inhibitors	PET/CT	10%	DR is associated with a clinical benefit of immunotherapy	Month 3

*DR, dissociated response; TTP, time-to progression; CT, Computed tomography; PET/CT, Positron emission tomography/computed tomograpy; NSCLC, Non-Small Cell Lung Cancer; EGFR, Epidermal growth factor receptor; EGFR-TKI, epidermal growth factor receptor-tyrosine kinase inhibitor; PD1, programmed cell death-1; PD-L1, programmed death-ligand 1*.

## Impact of the Primary Cancer Type and Treatment Type

A dissociated response can be observed with chemotherapies, targeted-therapies or immunotherapies, but its frequency varies across treatment types and primary cancer types. It seems more common in cancers with heterogeneous molecular profiles between metastases, such as breast cancer (16), and has recently been described with treatments targeting the immune response (Table 1). Surprisingly, few studies have evaluated the prognostic significance of such an atypical pattern.

### Chemotherapies + Targeted Therapies in Solid Cancers

#### Metastatic Breast Cancer (mBC)

In mBC, monitoring the treatment response with CT scan is hampered by the high frequency of bone metastases (70%) for which the apparent size does not necessarily change with treatment response (17, 18). The Response Evaluation Criteria in Solid Tumors (RECIST V1.1) specify that only lytic or mixed bone lesions with soft tissue components can be considered as measurable lesions (19). Because FDG PET/CT does not have this limitation, mBC is one of the first solid cancers for which PET has been routinely used to assess response. This is also the first metastatic setting in which the dissociated response was described. In 2010, Huyge et al. showed the intra-individual variability of the PET/CT metabolic response among lesions in bone-dominant metastatic breast cancer patients (12). These women were treated with different systemic therapies: chemotherapy (78%), hormone therapy (35%), anti-HER2 targeted therapy (4%). The metabolic response was analyzed according to the European Organization for Research and Treatment of Cancer (EORTC) criteria. Dissociated metabolic response occurred in 48% of patients, concerning mostly bone lesions, and tended to be associated with a better outcome than homogeneous non-response (*p* = 0.07). This result may suggest that, in case of dissociated disease evolution, the prognosis will depend on the number, the localization and the intrinsic aggressivity of the progressing lesion. This is, to our knowledge, the only available study on this topic in mBC.

#### Non-small-cell Lung Cancer (NSCLC)

In 2014, using CT scan, Lee et al. published a retrospective study including 68 patients with NSCLC who received second line EGFR-TKIs after a progression under systemic treatment (11). They observed that 32% of patient showed a dissociated response, and that this pattern was more frequent than homogeneous progression (19% of patients). Dong et al. published a larger retrospective study in 2017, including 246 consecutive patients with NSCLC and a response assessment with FDG PET/CT (3). The overall incidence of dissociated response was 21.5% and tended to occur more often in patients with advanced NSCLC (IIIB-IV) than those with earlier disease (I-IIIA) (30.0 vs. 5.8%, *p* < 0.001) and in patients treated with targeted therapies (EGFR-TKI) compared to those treated with chemotherapy (47.2 vs. 28.0%, *p* = 0.008). A dissociated response was an independent unfavorable prognostic factor for PFS (*p* = 0.04) and OS (*p* = 0.006) compared to patients with homogeneous evolution (homogeneous response or non-response). Interestingly, patients having a dissociated response were further categorized into those with “efficacious” dissociated response (i.e., only local PET-based disease progression and few clinical symptoms) and those with “inefficacious” dissociated response (the other patients). Most of the patients with efficacious dissociated response (65%) maintained prior regimens, with or without local intervention, whereas most patients with inefficacious dissociated response (63%) switched to next-line regimens. Compared to patients with an inefficacious dissociated response, the patients with an efficacious one showed a significant improvement in progression-free survival (9.4 vs. 3.8 months; *p* = 0.012) and overall survival (26.5 vs. 9.5 months; *p* = 0.027). This result underlines the need to recognize different patterns of dissociated response in NSCLC to improve outcome prediction for these patients.

#### Metastatic Colorectal Cancer (mCRC)

In a prospective multicentric study including 92 patients with a mCRC treated with a combination of sorafenib and capecitabine, a dissociated response was observed in one third of patients on interim FDG PET/CT (7). The presence of at least one metabolically non-responding lesion was associated with a poor outcome compared to patients without any metabolically non-responding lesions. But no prognostic difference was observed between patients with a dissociated response and patients with a homogeneous non-response. Therefore, the study concluded that the presence, but not the number, of non-responding lesions was the most important prognostic determinant. In a smaller study, only including nine patients with advanced KRAS wild-type mCRC treated with anti-EGFR therapy, a dissociated response was observed in nearly half of the patients (10).

#### Melanoma

In a Phase I monocentric trial evaluating the metabolic response of 23 patients with a BRAF mutant metastatic melanoma treated with dabrafenib, an heterogeneous PET response was observed in 26% of patients on the first interim PET performed 2 weeks after treatment initiation, and was associated with a shorter time-to-progression compared to homogeneous response (9). No patients with homogeneous lesion progression was observed which is consistent with the high level of activity of dabrafenib in these patients. It is worth noting that the more commonly reported measure of ΔSUVmax (metabolic change in the hottest lesion) was not found to be a prognostic biomarker in this study.

### Immunotherapies in Solid Cancer

Immunotherapy with immune checkpoints inhibitors represent a recent breakthrough in the treatment of various metastatic cancers, showing a benefit in overall survival (OS) across a broad range of cancer types (20–23). Indeed, a subset of patients with metastatic cancer can demonstrate a clinical benefit that can last several years even after stopping the treatment (20–23). Nonetheless, most patients do not exhibit response to immunotherapy and identifying patients that will benefit from immunotherapy as early as possible remains a crucial issue.

Because tumor shrinkage is not the unique pattern of tumor response anymore, immunotherapy has raised new challenges in the evaluation of tumor response, as much with CT than with PET/CT. Indeed, some responding-patients can have a transient increase in tumor burden and metabolism, or appearance of new lesions, followed by a delayed response or stability. This specific immune-related response pattern is termed ‘pseudo-progression' (PsPD) and is explained by the immune infiltration of tumors that can both induce a morphologic and metabolic increase of lesions (24, 25). This has been integrated in new immunotherapy-adapted criteria for CT to maintain the treatment in patients beyond a first imaging progression: the iRECIST (26). When using FDG PET, new immune-related response criteria for solid tumors have also been proposed, but without any consensus.

Beyond pseudo-progression, three recent studies have shown that a dissociated response is another atypical evolutive pattern of response to immunotherapy in advanced NSCLC with prognostic significance. Dissociated response is defined as the coexistence of responding and non-responding lesions within the same patient. Using CT imaging, Tazdait et al. retrospectively evaluated 160 patients with NSCLC treated with anti-PD1/PD-L1 drugs (13). They applied different morphologic imaging criteria (RECIST V1.1, iRECIST, irRECIST) and found, on the first CT evaluation, 7.5% of patients exhibiting a dissociated response. Atypical patterns (pseudo-progression + dissociated response) were associated with a better overall survival than true progressions. Another retrospective study including 62 NSCLC patients also observed a dissociated response in 9.2% of patients treated with PD-1/L1 inhibitors, and confirmed the improved OS associated to this pattern compared to homogenous progression (14.0 vs. 6.6 months) (14).

Using FDG PET/CT, our team recently published a prospective study including 50 patients with NSCLC treated with pembrolizumab/nivolumab and demonstrating that 12% of the population had a pseudo-progression and 10% had a dissociated response (15). Unlike what had been done in previous studies, the dissociated response was not defined on the first PET/CT evaluation showing a PERCIST disease progression, but on the subsequent confirmatory PET evaluation performed a few weeks later (3 months after treatment initiation). Because all these patients with dissociated response had a preserved clinical status and a limited number of progressive lesions, the patients' oncologists decided to maintain the therapy. A 6-months clinical benefit of immunotherapy was reached for all of them. Thus, it's worth noting that a dissociated response, contrary to pseudo-progression, is not only described on the first PET exam showing a metabolic tumor progression but can be described at later time-points of disease progression.

To sum up, a dissociated response appears to be a common evolutive pattern during immunotherapy (around 10% of treated patients), as frequent as the well-described pseudo-progression pattern. As Tazdait et al. have mentioned, this profile can be difficult to identify when using the conventional RECIST assessment, and requires a deep analysis of CT images (13), whereas this pattern can be easier to identify with PET/CT due to its ability to analyze the whole pool of lesions with great sensitivity. These studies also underlined that a dissociated response corresponds to a sign of treatment efficacy rather than failure, with a favorable outcome compared to homogeneous progression. Yet the prognostic value of the other atypical evolutive pattern, i.e., pseudo-progression, still needs to be explored. Furthermore, the best time point to assess these evolutive patterns will need to be defined: on the first or on the subsequent imaging evaluation?

## Current Strategies and Future Directions

Studying tumoral heterogeneity requires assessing the response of the whole baseline metastatic tumor load without restriction in number of lesions nor sites. However, both morphological (RECIST V1.1, iRECIST…) and metabolic (EORTC, PERCIST) response criteria only consider a limited number of operator-selected target lesions (19, 26, 27). Thus, the clinical scenario of a dissociated response of a single metastatic lesion has currently neither been integrated to morphological nor to metabolic criteria of response. More concerning, a proper, consensual name has not even been given to this pattern yet, as it can be referred to as “mixed response,” “heterogeneous response,” or “dissociated response.”

There is no clear consensus concerning the cut-off of changes in lesion size or metabolism (SUV) to define response or progression at a lesion level and therefore to define dissociated response at a patient level. In our point of view, dissociated response on CT exam should be inspired of RECIST V1.1 criteria and defined as a concomitant relative decrease in size >30% in some lesions and relative increase in size >20% in others (and/or presence of new lesions). On PET/CT, dissociated response definition should be inspired by PERCIST criteria and defined as a concomitant relative decrease >30% in some tumor lesions metabolism (ΔSUV) and relative metabolic increase >30% in others (and/or new hypermetabolic lesions).

The low consideration for this evolutive pattern may be because the response assessment is usually reported dichotomously by the radiologist (progression vs. response) to ease the clinician that needs to take a decision to continue or change the treatment. Although a specific prognostic value of dissociated response has been underlined in some types of solid cancers, it was always considered as an unfavorable pattern of response. Dissociated response was therefore included in the “Progressive Disease” category of RECIST/PERCIST, based on the assumption that “one progressive lesion is enough to define progression.”

**Figure 2:**
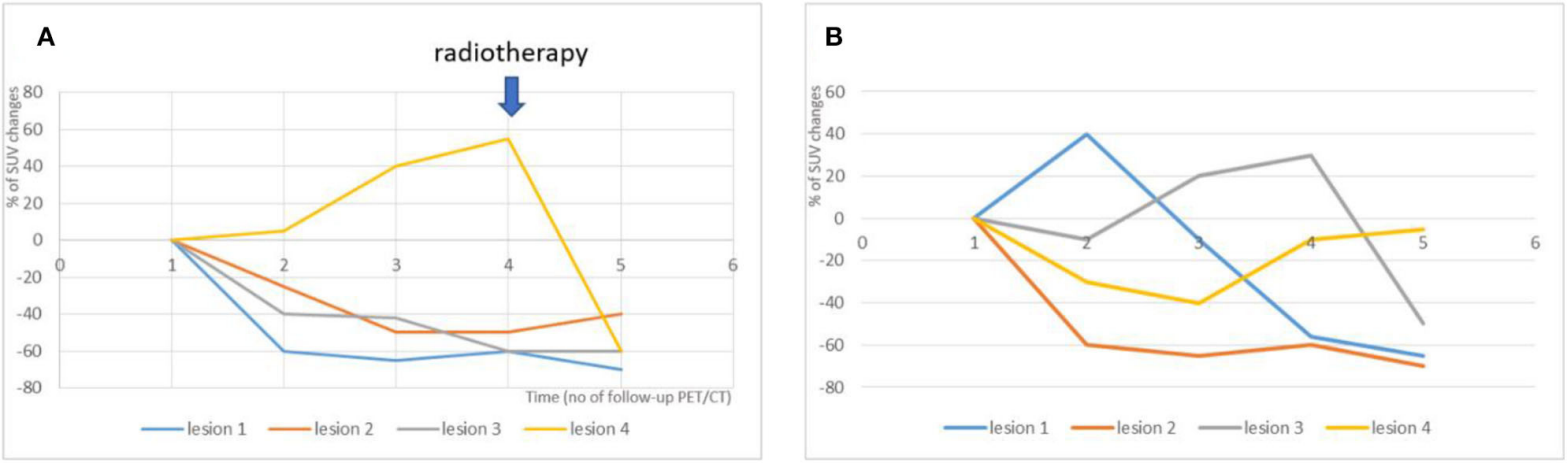
Illustration of two different dissociated response patterns in patients with NSCLC treated with immunotherapy and subsequent clinical management. **(A)** A continuous progression of one isolated metastatic lesion was observed across successive PET/CT exams (yellow line), while the 3 other metastatic lesions were responsive to treatment (orange, blue and gray lines). The Multidisciplinary Tumor Board decided to continue immunotherapy with concomitant local radiotherapy on the progressive lesion (blue arrow). **(B)** Transient immune activation “one after the other” of various metastatic lesions was observed. This pattern is very close to the standard pseudo-progressive pattern. In this pattern, the Multidisciplinary Tumor Board decided to maintained immunotherapy, with no need of local treatment.

With immune checkpoints inhibitors, the paradigm is quickly evolving: dissociated response is becoming a favorable prognostic pattern that absolutely needs to be distinguished from true progression. When a dissociated response to immunotherapy is observed, the continuation of the immune checkpoint inhibitors can provide a durable response (15). In our experience, different patterns of dissociated response can be observed in the immunotherapy setting (Figure 2):

- the continuous progression of oligo-metastatic lesions across successive exams, while the rest of the metastatic disease is under control. Because these few lesions show consistent resistance to immune therapies in consecutive exams, adding local ablative treatments to the growing lesions while pursuing the immune check-points inhibitors, may be a way to restore the prognosis.
- transient immune activation “one after the other” of various metastatic lesions, a pattern that is very close to the standard pseudo-progressive pattern. In this pattern, immunotherapy should be maintained, with no need of local treatment.

Thus, a dissociated response requires a specific categorization and should be captured in the future immunotherapy-adapted guidelines and criteria for CT and PET/CT, as is pseudo-progression in iRECIST. Further prospective works will be necessary to study the frequency and prognostic significance of the different dissociated patterns and optimize the best clinical management for each of them.

## Data Availability Statement

The original contributions presented in the study are included in the article/supplementary material, further inquiries can be directed to the corresponding author/s.

## Author Contributions

All authors listed have made a substantial, direct and intellectual contribution to the work, and approved it for publication.

## Conflict of Interest

The authors declare that the research was conducted in the absence of any commercial or financial relationships that could be construed as a potential conflict of interest.
